# Bio-Sensing of Cadmium(II) Ions Using *Staphylococcus aureus*^†^

**DOI:** 10.3390/s111110638

**Published:** 2011-11-08

**Authors:** Jiri Sochor, Ondrej Zitka, David Hynek, Eva Jilkova, Ludmila Krejcova, Libuse Trnkova, Vojtech Adam, Jaromir Hubalek, Jindrich Kynicky, Radimir Vrba, Rene Kizek

**Affiliations:** 1 Department of Chemistry and Biochemistry, Faculty of Agronomy, Mendel University in Brno, Zemedelska 1, CZ-613 00 Brno, Czech Republic; E-Mails: sochor.jirik@seznam.cz (J.S.); zitkao@seznam.cz (O.Z.); d.hynek@email.cz (D.H.); evajil@centrum.cz (E.J.); lidakrejcova@seznam.cz (L.K.); libuse@chemi.muni.cz (L.T.); vojtech.adam@mendelu.cz (V.A.); hubalek@feec.vutbr.cz (J.H.); 2 Central European Institute of Technology, Brno University of Technology, Technicka 3058/10, CZ-616 00 Brno, Czech Republic; E-Mail: vrbar@feec.vutbr.cz (R.V.); 3 Lead and Cadmium Initiatives, United Nations Environment Program, Faculty of Agronomy, Mendel University in Brno, Zemedelska 1, CZ-613 00 Brno, Czech Republic; 4 Department of Chemistry, Faculty of Science, Masaryk University, Kotlarska 2, CZ-611 37 Brno, Czech Republic; 5 Research Centre for Environmental Chemistry and Ecotoxicology, Faculty of Science, Masaryk University, Kotlarska 2, CZ-611 37 Brno, Czech Republic; 6 Department of Microelectronics, Faculty of Electrical Engineering and Communication, Brno University of Technology, Technicka 10, CZ-616 00 Brno, Czech Republic; 7 Department of Geology and Pedology, Faculty of Forestry and Wood Technology, Mendel University in Brno, Zemedelska 1, CZ-613 00 Brno, Czech Republic; E-Mail: jindrich.kynicky@mendelu.cz (J.K.)

**Keywords:** biosensor, cadmium, *Staphylococcus aureus*, metabolic activity, metabolome, microbiome, electrochemistry, voltammetry, Brdicka reaction, spectrophotometry, high performance liquid chromatography with electrochemical detection

## Abstract

Cadmium, as a hazardous pollutant commonly present in the living environment, represents an important risk to human health due to its undesirable effects (oxidative stress, changes in activities of many enzymes, interactions with biomolecules including DNA and RNA) and consequent potential risk, making its detection very important. New and unique technological and biotechnological approaches for solving this problems are intensely sought. In this study, we used the commonly occurring potential pathogenic microorganism *Staphylococcus aureus* for the determination of markers which could be used for sensing of cadmium(II) ions. We were focused on monitoring the effects of different cadmium(II) ion concentrations (0, 1.25, 2.5, 5, 10, 15, 25 and 50 μg mL^−1^) on the growth and energetic metabolism of *Staphylococcus aureus*. Highly significant changes have been detected in the metabolism of thiol compounds—specifically the protein metallothionein (0.79–26.82 mmol/mg of protein), the enzyme glutathione S-transferase (190–5,827 μmol/min/mg of protein), and sulfhydryl groups (9.6–274.3 μmol cysteine/mg of protein). The ratio of reduced and oxidized glutathione indicated marked oxidative stress. In addition, dramatic changes in urease activity, which is connected with resistance of bacteria, were determined. Further, the effects of cadmium(II) ions on the metabolic pathways of arginine, β-glucosidase, phosphatase, N-acetyl β-d-glucosamine, sucrose, trehalose, mannitol, maltose, lactose, fructose and total proteins were demonstrated. A metabolomic profile of *Staphylococcus aureus* under cadmium(II) ion treatment conditions was completed seeking data about the possibility of cadmium(II) ion accumulation in cells. The results demonstrate potential in the application of microorganisms as modern biosensor systems based on biological components.

## 1. Introduction

**Scheme 1.**
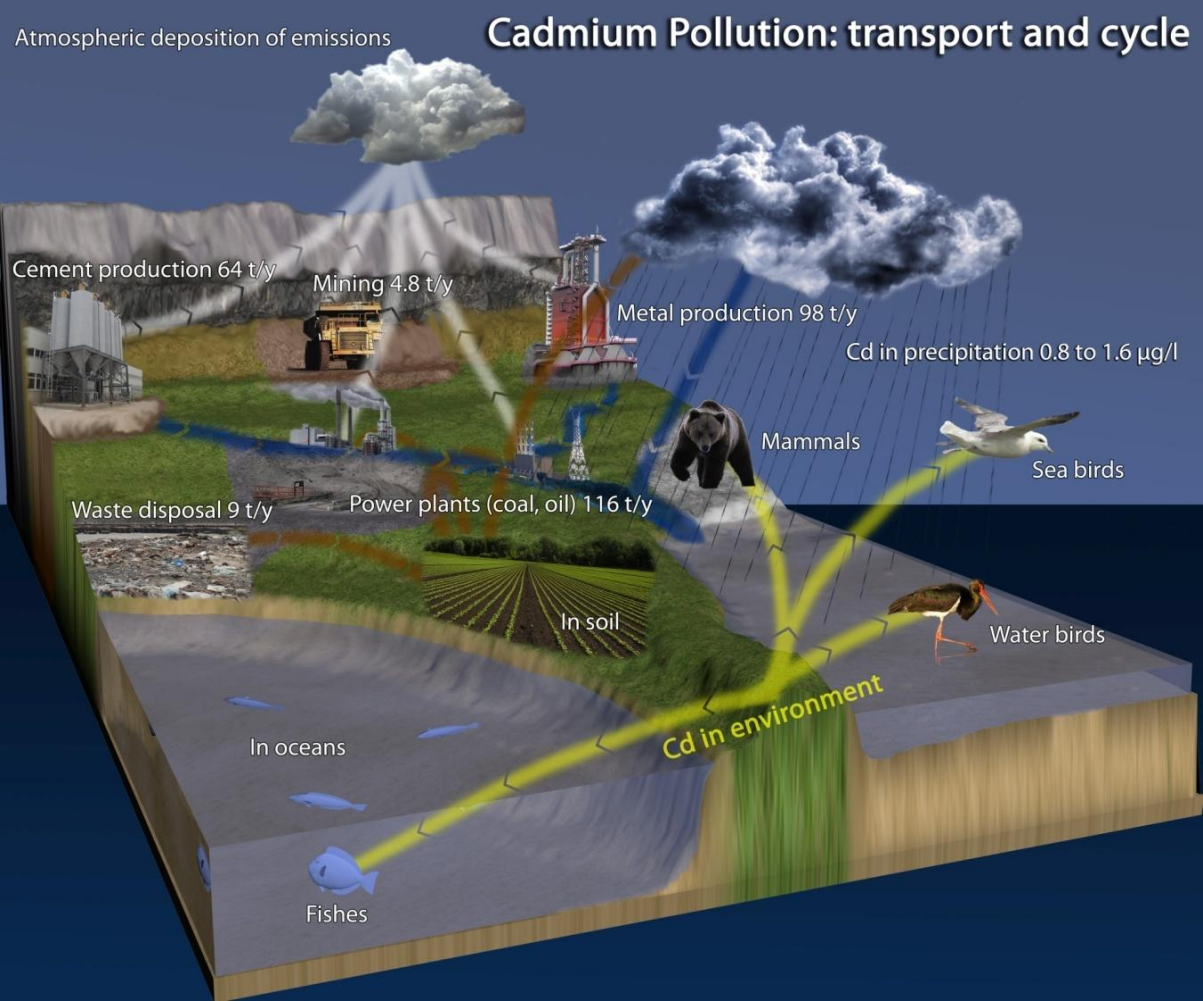
Cadmium pollution—transport and cycle. Adapted according to UNEP Lead and Cadmium activities.

Environmental pollution by xenobiotics is increasingly becoming a global issue. In connection with the growing ecosystem contamination by xenobiotics, it is therefore increasingly important to monitor their presence and promptly assess potential risks to humans [1]. The United Nations Environment Programme (UNEP) aims at monitoring and removing cadmium from the environment [2]. Over the last 15 years The Comprehensive Environmental Response, Compensation, and Liability Act (CERCLA) has permanently listed Cd as No. 7 (out of 275 species) in its priority list of hazardous materials [3]. Cadmium’s fate in the environment is shown in Scheme 1. Cadmium in its elemental form is a soft, silver-white metal, which occurs with other elements in the Earth’s crust with average content of 0.13–0.2 g t^−1^ in the lithosphere. This element is naturally found in air, water resources and soil as complex oxides, sulphides, and carbonates in zinc, lead, and copper ores [4]. Mining of iron and zinc ores, the burning of fossil fuels, plastics, dyes or road transport constitute the main sources of environmental cadmium pollution, and therefore the routes whereby it can enter the human food chain [5]. In mining global cadmium production increased during the period from 1970 to 2004 from about 17,000 tonnes to about 22,000 tonnes. Over the last 15 years, global consumption has remained relatively constant, at around 20,000 tonnes. Improperly disposal of batteries is another source of cadmium pollution [6]. In the atmosphere cadmium is mainly emitted to the atmosphere in particulate form. From combustion sources, cadmium may, however, be emitted partly as elemental gaseous cadmium, but as it is cooled, this cadmium is also quickly bound to particulate matter, so atmospheric transport of cadmium is governed by aerosol (particle) transport mechanisms.

**Figure 1.**
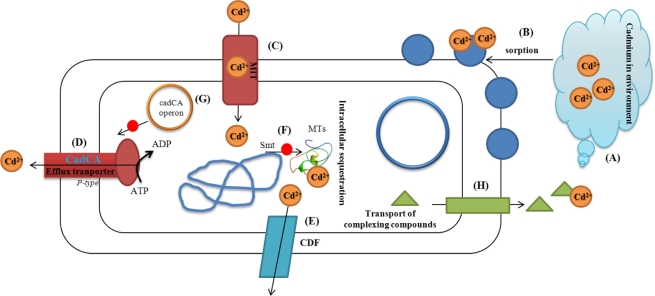
Overview of bacteria cadmium interaction. (**A**) Cd(II) ions occur in environment (soil, water, biota); (**B**) Sorption of Cd(II) on the surface of bacterial wall (protein, cyrbohydrates); (**C**) ion transporter (metal transporting system—MIT, which enable Cd(II) to enter cell; (**D**) Efflux transporter: CadCA protein, which is a P-type ATPase; (**E**) Slow efflux is catalysed by cation-diffusion facilitator CDF; (**F**) Intracellular sequestration: Smt metallothionein locus on bacterial chromosome transcript and translate metallothionein, which binds Cd(II) and, thus, protects a cells against adverse effects of these ions; (**G**) cadCA cadmium resistance operon *in Staphylococcus aureus* on plasmid transcript and translate CadCA (D). (G) Export of chelating compounds (organic acids), which interact with Cd(II) ions directly in outer space to form complexes, which can not enter the cell. Scheme was adopted and modified according to the following papers [11,13,19].

Quite extensive data sets of cadmium concentrations in the water column exist for specific locations in the world’s oceans and for different years over the last two to three decades. Through the literature search performed for this review, however, no examples of modelling or other quantification of the general horizontal transport of cadmium—or any other heavy metals—with ocean currents have been identified. Only two examples of quantification of the exchange of heavy metals (lead and cadmium) with ocean currents between one specific ocean, the Arctic Ocean, and neighbouring oceans was identified. These examples suggest that ocean transport may be an important pathway. In addition, the presence of cadmium in ammonium and phosphorus fertilizers is other important entry route of cadmium into the soil [7,8]. The chemistry of cadmium is to a great extent controlled by pH. Cadmium may be adsorbed on clay minerals, carbonates or hydrous oxides of iron and manganese or may be precipitated as cadmium carbonate, hydroxide, and phosphate. Under acidic conditions cadmium solubility increases, and very little adsorption of cadmium by soil colloids, hydrous oxides, and organic matter takes place. Both toxicity and bioavailability of cadmium are influenced by soil characteristics. Although cadmium is ranked as non-essential heavy metal, it is already toxic to plants, animals and humans at low doses and acts as a cumulative poison [9,10]. Some cadmium compounds are relatively water soluble, mobile in soil and bioavailable, depending on the water and soil chemistries. It tends to bioaccumulate in organs such as the kidney and liver of vertebrates, but aquatic invertebrates and algae can also build up relatively high concentrations. Effects on birds and mammals are mainly due to kidney damage. In sea birds and marine mammals in particular, cadmium accumulates to relatively high levels. Microorganisms are very prone to such accumulation, however, this phenomenon makes it possible to use some microorganisms as a biosensor for detection of selected substances contaminating the environment [10]. The mechanisms of metal accumulation by microorganisms are summarized in Figure 1: (1) metal resistance of microbes is accomplished by intra- and extracellular mechanisms; (2) metals can be excreted via efflux transport systems; (3) sequestering compounds of the cytosol can bind and detoxify metals inside the cell; (4) the release of chelators into the extracellular milieu leads to bound and fixed metals; (5) the structure of the cell envelope is prone to bind large amounts of metals by sorption thus preventing influx [11]. A great number of heavy metal resistant bacteria such as *Cupriavidus metallidurans* and others, is known to possess efflux transporters that excrete toxic or overconcentrated metals [12–15].

For metal ions to have physiological or toxic effects, they must enter the bacterial cell. Microbial uptake systems have to be tightly controlled to be able to differentiate between structurally very similar metal ions. Microorganisms use fast and unspecific uptake systems driven by the chemiosmotic gradient across the cytoplasmic membrane of bacteria. Membrane transport of cadmium(II) ions at *Staphylococcus aureus* has been summarized in [13,16–18]. The toxicity of heavy metal ions inside the cell may occur through the displacement of essential metals from their native binding sites or through ligand interactions. Especially heavy metal cations with high atomic numbers, e.g., Hg(II), Cd(II) and Ag(I), tend to bind SH groups [12,13,19]. By binding to SH groups, the heavy metal ions may inhibit the activity and/or the functioning of sensitive enzymes. Cations can also be segregated into complex compounds by thiol-containing molecules while on the other hand some heavy metal ions may be reduced to less toxic oxidation states [13]. A metal compound that can be reduced should be able to diffuse out of the cell. Most divalent heavy metal ions are accumulated within the cells by the fast and unspecific CorA (metal transport system) Mg(II) transport system [13]. Accumulation of Cd(II) in Gram-positive bacteria leads to the expression of the CadA resistance system (Figure 1), which is located on plasmid p1258 and related plasmids [12,20–22]. Cation efflux is catalysed by the CadA protein, which is a P-type adenosine triphosphatase (ATPase). ATP serves as a source of energy for CadA-catalysed cadmium transport [17]. It has also been found that amplification of Smt metallothionein (MT) locus increases cadmium resistance and deletion of Smt decreases resistance [23].

A biosensor is an analytical device comprising a biological recognition element (e.g., enzyme, receptor, DNA, antibody, or microorganism) in intimate contact with an electrochemical, optical, thermal, or acoustic signal transducer that together permit analyses of chemical properties or quantities [24].

Microorganisms are suitable as biosensors thanks to their fast “*in situ*” analysis because of rapid bacterial cell growth and dividing, adaptability, resilience, and their metabolic activity [25–34]. In terms of construction of biosensors, microorganisms are among the most promising biological materials, because each cell represents an independent individual, and is therefore usually more resistant and more durable as compared with cellular components and tissues organisms, which was experimentally demonstrated [35]. Other advantages include the wide range of substances which cause a response, because of convergent metabolic pathways [33,36]. Generally, bacterial biosensors most frequently use electrochemical detectors as the amperometric [37,38], potentiometric [39], or conductometric [40] methods or optical detectors measuring bioluminescence [41], fluorescence [42] and/or colorimetric sensing [43]. Microbial biosensors based on the detection of changes in pressure [44] or respiration [45] are less widely used. Microbial biosensors are well reviewed by Lei *et al*. [46]. Microorganisms such as biosensors are widely used in healthcare, in control of foodstuffs [47], agriculture [36] or the environment [48]. Using such biosensors can determine a wide range of organic compounds such [38], heavy metals [41,49] and other types of xenobiotics [36,39,43,50]. Microbial biosensors based on genetically modified microorganisms are other well developed area [51,52]. Bioreporters belong to the most promising, whose design uses two key genes that are responsible for producing a measurable signal and analyte-specific recognition and subsequent activation of reporter genes [53]. Genes are based on contact with a chemical compound or changing physico-chemical conditions (pH, temperature, osmotic pressure, electric potential), activated when the activation leads to generation of a specific and easily measurable signal [54,55]. The use of bioreporters can be particularly advantageous in the detection of contaminated sites, where mixtures of different compounds occur, usually because bioreporters detect groups of related substances rather than individual chemicals [56].

The aim of this study was to demonstrate the principle and possibilities of metabolic signals (changes in growth, changes in the content of cadmium(II) ions, glutathione, metallothionein, free thiol moieties, activity of glutathione-S-transferase, total protein content, metabolism of sucrose, lactose, fructose, mannose, trehalose, N-acytyl-d-glucosamine, mannitol, urease, phosphatase and arginine) of *Staphylococcus aureus* as a biosensor to monitor cadmium(II) ions.

## 2. Experimental Section

### 2.1. Chemicals

All chemicals used (of ACS purity) were purchased from Sigma Aldrich (USA) unless noted otherwise. Cd(NO_3_)_2_ was used in our experiments as a source of cadmium(II) ions. Acetate buffer of pH 5 was prepared with 0.2 M acetic acid and 0.2 M sodium acetate and diluted with water and used as a supporting electrolyte. High purity deionised water (Milli-Q Millipore 18.2 MΩ/cm, Bedford, MA, USA) was used in the study.

### 2.2. Cultivation of Bacterial Strains

*Staphylococcus aureus* (NCTC 8511) was obtained from the Czech Collection of Microorganisms, Faculty of Science, Masaryk University, Brno, Czech Republic. Strains were stored as a spore suspension in 20% (*v*/*v*) glycerol at −20 °C. Prior to use in this study, the strains were thawed and the glycerol was removed by washing with distilled water. The bacterial strain was incubated in the presence of cultivation medium (meat peptone 5 g L^−1^, NaCl 5 g L^−1^, bovine extract 1.5 g L^−1^, yeast extract 1.5 g L^−1^ (HIMEDIA, Mumbai, India)), sterilized MiliQ water with 18 MΩ) at 600 rpm and 37 °C in Incubator Hood TH 15 (Edmund Buhler GmbH, Hechingen, Germany). pH of the cultivation medium was adjusted at 7.4 before sterilization. Sterilization was carried out at 121 °C for 30 min. in sterilizer (BMT, Brno, Czech Republic). Grown bacterial culture was diluted by cultivation medium to OD_600_ = 0.1 prior to use in the following experiments. The prepared medium (10 mL) was pipetted into 25 mL flasks and cadmium(II) ions (0, 1.25, 2.5, 5, 10, 15, 25 and 50 μg mL^−1^) were added.

### 2.3. Growth Curves

Solution containing bacteria, cultivation medium and various concentrations of cadmium(II) ions was mixed and pipetted into plastic tubes (3 mL) (AnalytikJena, Jena, Germany). Subsequently, a SPECORD 210 device (AnalytikJena) was used for measuring of the solution absorbance at a wavelength of 600 nm every 30 min for 24 h. A carousel for eight samples was used. All measurements were done in five replicates. The resulting absorbance were averaged and recalculated to the control variant, which represented 100%. Cuvette area was thermostated throughout the experiment to 37 °C (F12/ED Julabo, Seelbach, Germany). The SPECORD device was controlled by the WinASPECT Version 2.2.7.0 program package (AnalyticJena).

### 2.4. Preparation of Biological Samples

#### 2.4.1. Spectrophotometric Measurements (Metabolic Parameters, Total Protein Content, Sulfhydryl Groups and Glutathione-S-Transferase) and Chromatographic Measurements (Reduced and Oxidized Glutathione)

The obtained cells were washed three times with phosphate buffer of pH 7. Weighed bacterial samples (approximately 0.1 g of fresh weight) were transferred to test-tube (2 mL) (Eppendorf, Hamburg, Germany), and liquid nitrogen was added. The samples were frozen to disrupt the cells. The mixture was prepared using a hand-operated ULTRA-TURRAX T8 homogenizer (IKA, Konigswinter, Germany) at 25,000 rpm for 3 min. The homogenate was transferred to a new test-tube. The mixture was further homogenised by shaking on a Vortex-2 Genie (Scientific Industries, New York, NY, USA) at 4 °C for 30 min. The homogenate was centrifuged (14,000 rpm) for 30 min at 4 °C using a Universal 32 R centrifuge (Hettich-Zentrifugen GmbH, Tuttlingen, Germany). Prior to analysis the supernatant was filtered through a membrane filter (0.45 μm Nylon filter disk, Millipore, Billerica, MA, USA).

#### 2.4.2. Electrochemical Measurement (Cadmium)

Content of cadmium(II) ions was determined in medium and in bacteria as free (without mineralization) and bound (samples were mineralized). *Medium*. A sample (2 mL) in a test tube was centrifuged at 1,500 rpm for 15 min. (Eppendorf). The obtained supernatant was pipetted and 500 μL of the supernatant was used for the determination of Cd(II) in cultivation medium. *Free cadmium(II) ions in bacteria*. The obtained cells were washed three times with phosphate buffer of pH 7. After the last wash 0.1 M phosphate buffer (pH 7.0, 1.5 mL) was added. The prepared sample was ultrasounded for 2 min at 40 W using a needle probe (Bandelin, Berlin, Germany). Homogenates were then vortexed for 5 min at 400 rpm (Genie, New York, NY, USA) and then centrifuged for 15 min at 16,000 rpm (Eppendorf) prior to electrochemical analysis. *Bound cadmium(II) ions in bacteria*. The obtained cells were washed three times with phosphate buffer of pH 7. To prepare the samples microwave digestion were used according to recently published papers [15,57,58]. Briefly, the mineralization of samples took place in a microwave system Multiwave3000 (Anton-Paar GmbH, Graz, Austria). A sample (10 mg of bacteria) was placed into MG5 glass vials and (i) 350 μL of nitric acid (65%, *w*/*w*) and 150 μL of hydrogen peroxide (30%, *w*/*w*) or (ii) 700 μL of nitric acid (65%, *w*/*w*) and 300 μL of hydrogen peroxide (30%, *w*/*w*) were added. Prepared samples were sealed and placed into a 64MG5 rotor (Anton-Paar GmbH). The rotor with the samples was inserted into the microwave system and the microwave digestion was carried out under the following conditions: power 50 W—10 min, power 100 W—30 min, cooling (power 0 W)—10 min, maximum temperature 80 °C. Sample preparation for subsequent electrochemical measurements was as follows: 100 μL mineralized sample was pipetted into Eppendorf tubes with 900 μL acetate buffer (pH = 5.00). A blank digestion was simultaneously carried out in the same way.

#### 2.4.3. Electrochemical Measurement (Metallothionein)

The obtained cells were washed three times with phosphate buffer of pH 7. Weighed bacterial samples (approximately 0.2 g of fresh weight) were transferred to test-tube (2 mL) (Eppendorf), and liquid nitrogen was added. The samples were frozen to disrupt the cells. The mixture was prepared using an ULTRA-TURRAX T8 hand-operated homogenizer (IKA) at 25,000 rpm for 3 min. The homogenate was transferred to a new test-tube and vortexed for 15 min at 4 °C (Vortex Genie). The supernatant was subsequently heat treated. The sample was kept at 99 °C in a thermomixer (Eppendorf, Hamburg, Germany) for 15 min. with occasional stirring, and then cooled to 4 °C. The denatured homogenates were centrifuged at 4 °C, 15,000 rpm for 30 min. (Eppendorf 5402). Heat treatment effectively denatures and removes high molecular weight proteins out from samples [59–61].

### 2.5. Determination of Metabolic Parameters

Determination of urease and phosphatase activities, and quantification of arginine, N-acetyl β-d-glucosamine, sucrose, trehalose, mannitol, maltose, mannose, lactose and fructose was performed on a Multiskan EX analyser (ThermoScientific, Waltham, MA, USA). For determination of activities and/or presence of all mentioned substances, kits purchased from the same company as analyser were used. The kits were used for on-line monitoring of these substances in living bacteria cultivated in the special well. Basic principles of measurements of the markers were as follows: urease: the enzyme urease hydrolyses urea to ammonia, arginine: l-argininedihydrolase hydrolyses l-arginine; phosphatase: phosphatase hydrolyses nitrophenylphosphate to inorganic phosphate and *p*-nitrophenyl, N-acetyl β-d-glucosamine, galactose, sucrose, trehalose, mannitol, mannose, lactose, fructose: they are used as a source of carbon and energy, during their decomposition acid reaction is formed and detected.

For measurements themselves, chemicals are pipetted in microplate wells. These plates also contained the above prepared bacterial solutions. Incubation was done at 37 °C (Thermostat, Biosan, Latvia). Samples were measured at a wavelength of 420 nm and 540 nm. Measurements were carried each 30 min for 24 h. The device was controlled by the Ascent Software Version 2.6 program package (ThermoScientific).

### 2.6. Determination of Total Protein Content, Sulfhydryl Groups and Glutathione-S-Transferase

Spectrophotometric measurements of total protein content, sulfhydryl groups and glutathione-S-transferase were carried using an automated chemical analyzer BS-200 (Mindray, Shenzhen, China). Reagents and samples were placed on cooled sample holder (4 ± 1 °C) and automatically pipetted directly into plastic cuvettes. Incubation proceeded at 37.0 ± 0.1 °C. Mixture was consequently stirred. The washing steps of pipetting needle with distilled water (18 mΩ) were done in the midst of the pipetting. The instrument was operated using the BS-200 software (Mindray).

#### 2.6.1. Determination of Total Proteins Content

Determination of total proteins content using Bradford method is described in the following paper [62]. Briefly, reagent Coomassie Brilliant blue G-250 (0.01% Coomassie Brilliant Blue G-250, 4.7% CH_3_CH_2_OH, 8.5% H_3_PO_4_, *v*/*v*) in volume of 190 μL was pipetted into cuvette. Further, sample (10 μL) was added. Mixture was incubated at 37 °C for 10 min. Absorbance was measured at 595 nm, reagent itself was used as a blank. Obtained values of absorbance (blank, mixture after 10 min long incubation) were used for determination of total proteins content. For calibration, bovine serum albumin (Sigma-Aldrich) was used.

#### 2.6.2. Determination of Sulfhydryl Groups

Ellman’s spectrophotometric method was used for determination of sulfhydryl (-SH) moieties [63]. Ellman’s reagent (277 μL, R1, 2 mM 5.5′-dithiobis(2-nitrobenzoic) acid (DTNB) in 50 mM sodium acetate CH_3_COONa) was mixed with sample (45 μL). Further, reagent R2 (33 μL, 1 M trisma base: CH_3_COOH) was added. Mixture was incubated at 37 °C for 10 min. Absorbance was measured at 405 nm. Values of absorbance of reagent R1 itself (blank) and mixture after 10 min. long incubation were used for determination of total-SH content.

#### 2.6.3. Determination of Glutathione-S-Transferase

The method is based on glutathione-S-transferase (GST) catalysed reaction between reduced glutathione (GSH) and GST substrate, 1-chloro-2,4-dinitrobenzene (CDNB), which has the broadest range of isozyme detectability (e.g., alpha-, mu-, pi- and other GST isoforms). Under certain conditions, the interaction between glutathione and CDNB is dependent on the presence of active GST. The GST-catalysed formation of GS-DNB produces a dinitrophenylthioether, which can be detected spectrophotometrically at 340 nm [64]. A 180 μL volume of reactants consisting of 2 mM CDNB and PBS (1.4 mM NaH_2_PO_4_, and 4.3 mM Na_2_HPO_4_, pH 7.4) (1:19, *v*/*v*, 37 °C) was added to sample in a plastic microtube. Further, 12.5 mM GSH (30 μL) in 0.1 M phosphate buffer (pH 7.4) was added. A wavelength of 340 nm was used for determination of GST activity.

### 2.7. Determination of Metallothionein

Differential pulse voltammetry (DPV) Brdicka reaction measurements were performed with a 747 VA Stand instrument connected to 746 VA Trace Analyzer and 695 Autosampler (Metrohm, Zofingen, Switzerland), using a standard cell with three electrodes and cooled sample holder (4 °C) according to protocol by Fabrik *et al*. [59]. A hanging mercury drop electrode (HMDE) with a drop area of 0.4 mm^2^ was the working electrode. An Ag/AgCl/3M KCl electrode was the reference and glassy carbon electrode was auxiliary electrode. GPES 4.9 supplied by software EcoChemie was employed for smoothing and baseline corrections of the obtained data. A supernatant sample (200 μL) was pipetted into electrochemical cell containing 1,800 μL of Brdicka supporting electrolyte and measured using DPV. The electrolyte containing 1 mM Co(NH_3_)_6_Cl_3_ and 1 M ammonia buffer (NH_3_(aq) + NH_4_Cl, pH = 9.6) was used and changed per one analysis. DPV parameters were as follows: initial potential of −0.7 V, end potential of −1.75 V, modulation time 0.057 s, time interval 0.2 s, step potential 2 mV, modulation amplitude −250 mV, Eads = 0 V. All experiments were carried out at a temperature of 4 °C (Julabo F12 cooler).

### 2.8. Determination of Cadmium by Differential Pulse Voltammetry

Electrochemical analyser was used for determination of Cd(II) [25,65–67]. The analyser (757 VA Computrace, Metrohm AG, Zofingen, Switzerland) employs a conventional three-electrode configuration with a hanging mercury drop electrode (HMDE) working electrode: 0.4 mm^2^, Ag/AgCl/3MKCl as reference electrode, and a platinum auxiliary electrode. The following setup assembled of automated voltammetric analysis is supplied by Metrohm. An autosampler (Metrohm 813 Compact Autosampler) performs the sequential analysis of up to 18 samples in plastic test tubes. For the addition of standard solutions and reagents, two automatic dispensers (Metrohm 765 Dosimat) are used, while two peristaltic pumps (Metrohm 772 Pump Unit, controlled by Metrohm 731 Relay Box) are employed for transferring the rinsing solution in the cell and for removing solutions from the voltammetric cell. Differential pulse voltammetric measurements were carried out under the following parameters: deoxygenating with argon 90 s; deposition potential −0.8 V; time of deposition 240 s; start potential −0.8 V; end potential 0.15 V; pulse amplitude 0.025 V; pulse time 0.04 s; step potential 5.035 mV; time of step potential 0.4 s.

### 2.9. Determination of Reduced and Oxidized Glutathione

Reduced (GSH) and oxidized (GSSG) glutathione was determined using high performance liquid chromatography with electrochemical detection (HPLC-ED) [68]. The chromatographic system consisted of two solvent delivery pumps operating in the range of 0.001–9.999 mL min^−1^ (Model 582 ESA Inc., Chelmsford, MA, USA), Zorbax eclipse AAA C18 (150 × 4.6; 3.5 μm particle size; Agilent Technologies, Santa Clara, CA, USA) and a CoulArray electrochemical detector (Model 5600A, ESA). The electrochemical detector includes three flow cells (Model 6210, ESA). Each cell consists of four working carbon porous electrodes, each one with auxiliary and dry Pd/H_2_ reference electrodes. Both the detector and the reaction coil/column were thermostated. The sample (20 μL) was injected using autosampler (Model 542 HPLC, ESA). Samples were kept in the carousel at 8 °C during the analysis. The column was thermostated at 32 °C. Mobile phase consisted of 80 mM TFA (A) and methanol (B). The compounds of interest were separated by the following linear gradient: 0 → 1 min (3% B), 1 → 2 min (10% B), 2 → 5 min (30% B), 5 → 6 min (98% B). Mobile phase flow rate of 1 mL/min, working electrode potential 900 mV. Time of analysis was 20 min.

### 2.10. Preparation of Deionised Water and pH Measurement

Deionised water was prepared using Aqual 25 reverse osmosis equipment (Sigma Aldrich, St. Louis, MO, USA). The deionised water was further purified by using apparatus MiliQ Direct QUV equipped with the UV lamp. The resistance was 18 MΩ. The pH was measured using pH meter Accurate 7110 (WTW inoLab, Weilheim, Germany).

### 2.11. Mathematical Treatment of Data and Estimation of Detection Limits

Mathematical analysis of the data and their graphical interpretation was realized by the Matlab (version 7.11) software. Results are expressed as mean ± standard deviation (S.D.) unless noted otherwise (EXCEL^®^). The detection limits (3 signal/noise, S/N) and quantification limits (10 S/N) were calculated according to Long and Winefordner [69], whereas N was expressed as standard deviation of noise determined in the signal domain unless stated otherwise. Accuracy, precision and recovery of cadmium(II) ions were evaluated with homogenates (bacterial samples) spiked with standards. Before extraction, 100 μL cadmium(II) ions standards and 100 μL water were added to bacterial samples. Homogenates were assayed blindly and cadmium(II) ions concentrations were derived from the calibration curves. Accuracy was evaluated by comparing estimated concentrations with known concentrations of heavy metals compounds. Calculation of accuracy (%Bias), precision (%C.V.), root mean square error (RMS error) and recovery was carried out as indicated according to Causon [70] and Bugianesi *et al*. [71]. Equilibrium diagrams plotted using sophisticated algorithms (MEDUSA program) were used for the construction of a distribution diagram of different cadmium chemical forms present in the supporting electrolyte [72,73]. The basic parameters, including equilibrium constants that are necessary for the calculation of distribution diagrams are in the program database. The program author is Ignasi Puigdomenech from the Inorganic Chemistry of Royal Institute of Technology, Stockholm, Sweden. The MEDUSA program is freeware and is available on http://www.kemi.kth.se/medusa.

## 3. Results and Discussion

Transport of heavy metal ions in bacterial systems is not clear and requires further research. Bacterial sensors for detecting toxic substances including heavy metal ions belong to the areas receiving considerable attention. The reason for the use of living organisms as a sensor is the fact that living “bio” part of detection system is possible to obtain information of the effect of toxic substance on a cell together with obtaining the information about type of pollutant and its concentration.

### 3.1. Electrochemical Detection of Cadmium(II) Ions

**Figure 2.**
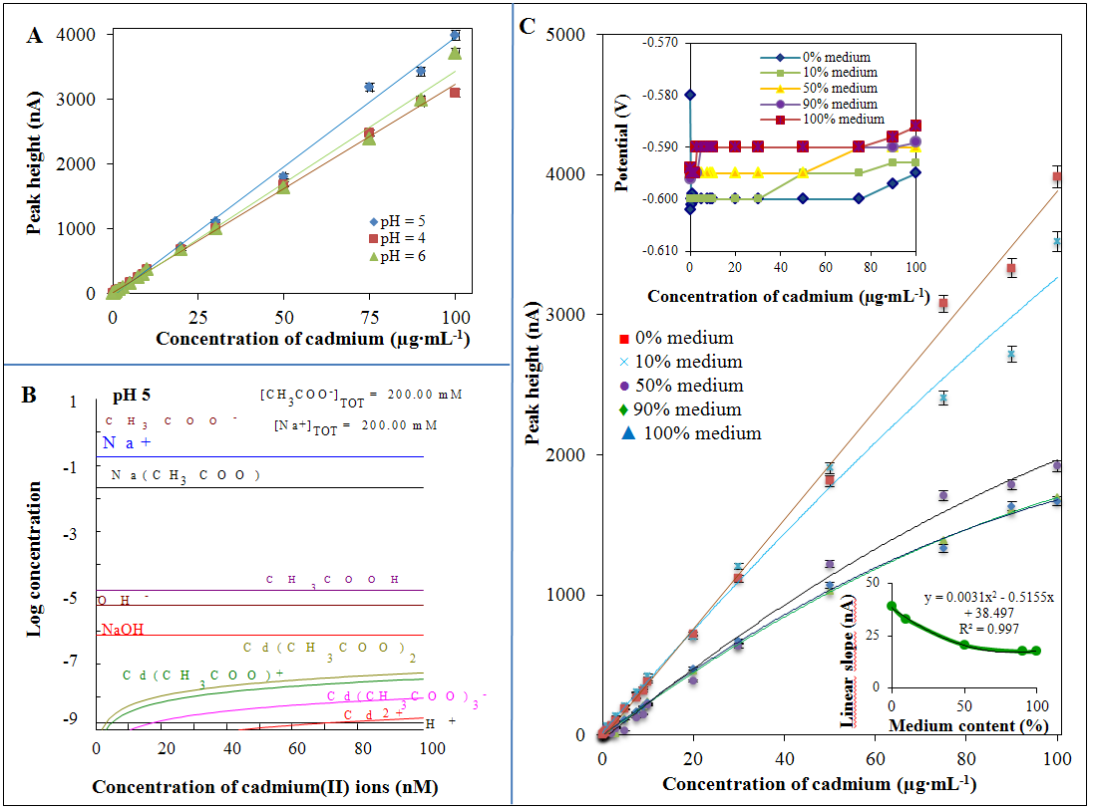
(**A**) Dependencies of peak height of cadmium(II) ions on their concentrations measured in the presence of acetate buffer pH 4, 5 and 6; (**B**) Mathematical modelling of cadmium(II) ions behaviour in the presence of 0.2 M acetate buffer, pH 5; (**C**) Changes in the peak height of cadmium(II) ions measured in the presence of 0, 10, 50, 90 and 100% (*v*/*v*) of cultivation medium in supporting electrolyte. Changes in (upper inset) potentials of the peaks and (upper inset) linear slopes with the increasing content of cultivation medium in supporting electrolyte as 0.2 M acetate buffer, pH 5. Differential pulse voltammetric measurements were carried out under the following parameters: deoxygenating with argon 90 s; deposition potential −0.8 V; time of deposition 240 s; start potential −0.8 V; end potential 0.15 V; pulse amplitude 0.025 V; pulse time 0.04 s; step potential 5.035 mV; time of step potential 0.4 s.

The mercury drop electrode has been using for the determination of heavy metals since the discovery of polarography by Prof. Heyrovsky. The method is very simple and low cost and the technology is also easy to automate [74]. In previously published papers, it is shown that cadmium ions can be detected well in acidic environments. Acetate buffer seems to be very suitable for this purpose, however, nature of the electrode process is not very clear [15,65]. To detect cadmium(II) ions, adsorptive stripping technique, in which heavy metals (cadmium) dissolve in mercury by forming amalgams, is used. Measurements are based on a preconcentration potential of −1.1 V (*vs*. Ag/AgCl) at a suitable deposition time, according to the following reaction: Cd^2+^ + ne^−^ → Cd(Hg) and reoxidizing step these metal into the solution: Cd(Hg) → Cd^2+^ + ne^−^ [75]. Typical dependencies of peak height of cadmium(II) ions on their concentrations measured in the presence of acetate buffer pH 4, 5 and 6 are shown in Figure 2(A). The obtained calibration curves were linear within tested concentration interval from 0.25 nM to 100 nM and are shown in Table 1.

In addition, the effect of mineralization procedure (microwave digestion) on the obtained calibration dependence was investigated. Two variants were tested: (a) 700 μL HNO_3_ + 300 μL H_2_O_2_; (b) 350 μL HNO_3_ + 150 μL H_2_O_2_. The data was verified by analysing the sample digest of cadmium(II) ions in independent repetitions (n = 3) and, moreover, the effect of above mentioned pH 4, 5 and 6 of acetate buffer was tested. Recoveries of cadmium(II) ions varied within the interval from 98.5 to 100.5% in the tested samples. The obtained calibration dependencies measured in samples mineralized by both above mentioned procedures were strictly linear for (a) R^2^ = 0.9968 and (b) R^2^ = 0.9942. There were observed marked differences in mineralization process expressed as slopes of the calibration curves as (a) 5.8 and (b) 4.4. The difference is significant and means a decrease of analytical signal for more than 20%. Therefore, the mineralization variant (a) (350 μL HNO_3_ + 150 μL H_2_O_2_) was used in the following experiments.

The distribution of individual forms of cadmium complexes (at a concentration from 0 to 100 nM) in the presence of 200 mM acetate buffer of pH 5 is shown in Figure 2(B). Given our objective to study the effects of cadmium(II) ions on bacterial culture of *S. aureus*, it was necessary to monitor the effects of interactions between cadmium(II) ions and the cultivation medium (its composition is described in the Experimental Section and is a very complex matrix, hardly defined). Components of the medium react with cadmium(II) ions very intensely, as it is shown in Figure 2(C). Interactions between cadmium(II) ions and ions of cultivation medium was studied after 60 min. of their mixing at 20 °C. Cadmium(II) ion peak height measured in the presence of 100% cultivation medium decreased for more than 70% compared to cadmium(II) ions standard. Addition of medium to supporting electrolyte led to a shift of the peak height for 10 mV to more positive potentials (in upper inset in Figure 2(C)). The apparent interaction between the components of medium and cadmium(II) ions was expressed by slopes of the measured dependencies (Figure 2(C)). It is obvious that the linear model is less suitable for describing of dependencies shown in Figure 2(C). Confidence coefficients are higher than 0.98 (Table 2). Using a polynomial mathematical models seems to be more convenient (Table 1) due to the fact that confidence coefficients are higher than 0.99. Slopes of the dependencies decreased with the increasing content of cultivation medium for 2 nA per 1% of the medium. Decrease in the slope was faster within the range of the addition of medium from 1 to 50%, at higher levels of medium there was moderate decrease in slope (see in bottom inset in Figure 2(C)). There is still not enough attention paid to the issue of the interactions between the components of cultivation medium and the actual concentration of compound of interest. These changes should be taken into account when one may monitor heavy metals in the environment.

### 3.2. Detection of Cadmium(II) Ions in Bacterial Samples

**Figure 3.**
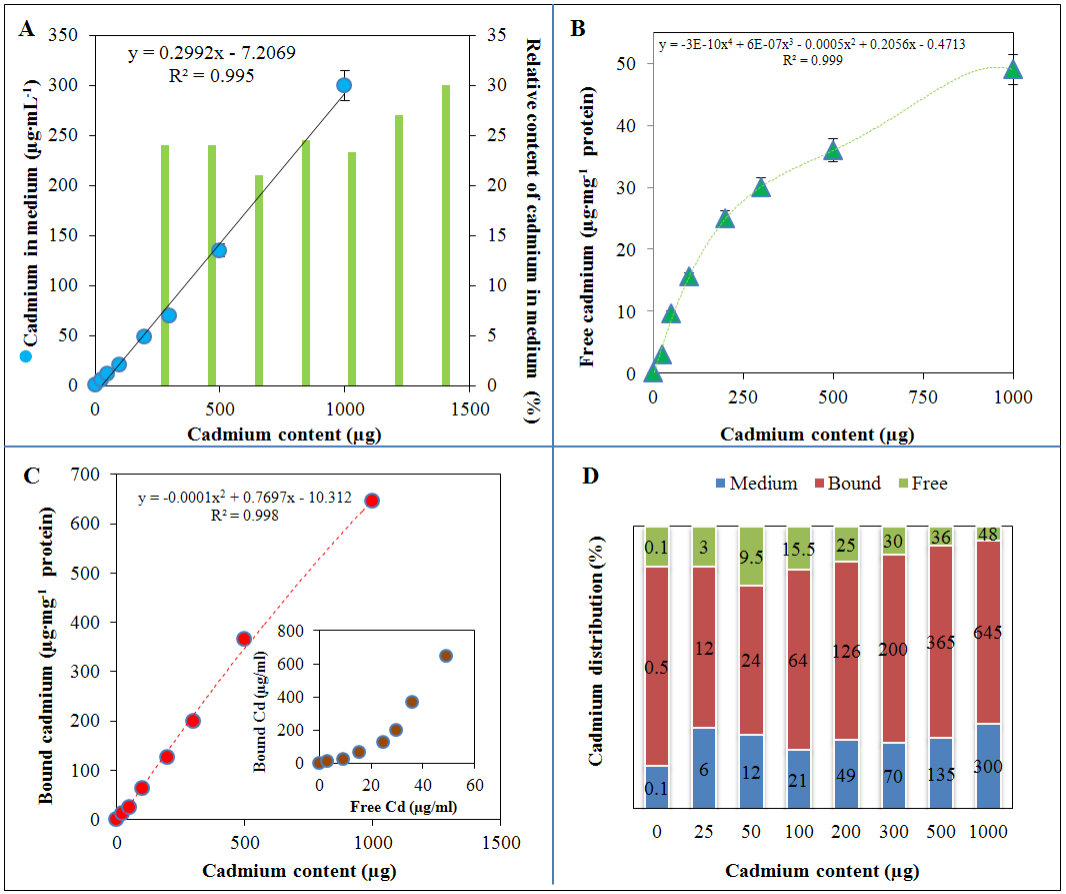
(**A**) Dependence of free Cd(II) content in cultivation medium on the applied concentration of cadmium(II) ions; green columns represent ratio as follows—(determined content of Cd(II)/given content of Cd(II)) × 100; (**B**) Free Cd(II) content in bacteria cells; (**C**) Content of bound Cd(II) in bacteria cells; in inset: correlation between free and bound Cd(II) content. Ratios between medium Cd(II) content, and bound and free Cd(II) content in bacterial cell (**D**). Other experimental conditions are given in Figure 2.

The interactions between cadmium(II) ions and *S. aureus* are not fully understood [18]. The resistance to cadmium connected with some plasmids and fragments called cadD and cadX regions was described [76]. A phenotypic association between reduced susceptibility to zinc and methicillin resistance in *S. aureus* was recently reported. The authors showed that czrC encodes zinc and cadmium resistance in CC398 MRSA isolates, and that it is widespread in both humans and animals. Thus, resistance to heavy metals such as zinc and cadmium may play a role in the selection of methicillin resistance in *S. aureus* [77]. These facts open up new possibilities for understanding the possible processes behind resistance of microorganisms to antibiotics. Sensor based monitoring can be beneficial to produce better technical ways of treating bacterial infections. In this study, after the above mentioned characterization and optimization steps, levels of cadmium(II) ions were determined in cultivation medium obtained after treatment of *S. aureus* with cadmium(II) ions as described in the Experimental section. The dependence of cadmium(II) ion content in cultivation medium is shown in Figure 3(A). As expected, the dependence is strictly linear, but the amount of free Cd(II) ranged between 23–30% of the total content of cadmium(II) ions added into the medium (green columns in Figure 3(A)).

In addition, contents of free and bound cadmium(II) ions in bacterial cells were determined. Figure 3(B) shows the change in free Cd(II) with the increasing applied concentration. The content of free Cd(II) increased up to dose of 250 μg Cd(II) in 25 mL of cultivation medium and, after that, the increase in free Cd(II) content was more gradual. The content of bound Cd(II) increased linearly with the applied concentration of Cd(II) (Figure 3(C)). The inset in Figure 3(C) shows the correlation between bound and free Cd(II) content. The observed dependence shows the two processes, which can be defined by the changes in the behaviour of cadmium(II) accumulation detected after exceeding of free Cd(II) content of 20 μg·mg^−1^ protein. Figure 3(D) summarizes the ratios between free and bound content of Cd(II) and content of Cd(II) in medium. The highest content Cd(II) was observed bound to the surface of *S. aureus* cell wall as it was expected. Moreover, Cd(II) content in bacterial cells decreased with the increasing applied concentration, which can be associated with triggering of a number of defence mechanisms against uptake of cadmium(II) ions.

### 3.3. Growth of S. aureus Treated with Cadmium(II) Ions

Growth of bacterial cells is among the important characteristics of the interactions between bacteria and toxic compounds. For this purpose, growth characteristics were monitored spectrophotometrically using OD measured at 605 nm.

**Figure 4.**
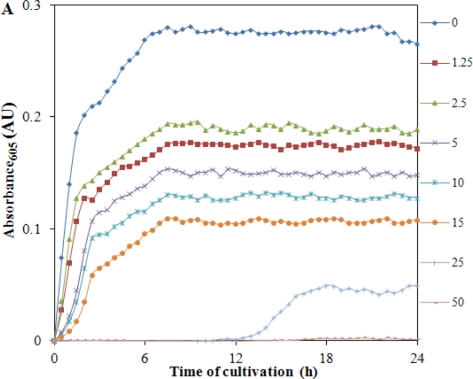

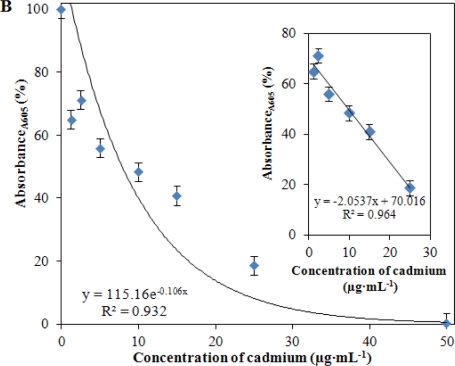
Spectrophotometric analysis of cadmium(II) ions treated *Staphylococcus aureus*. (**A**) Dependencies of growth of bacteria on the applied concentration of cadmium(II) ions (0, 1.25, 2.5, 5, 10, 15, 25 and 50 μg·mL^−1^) monitored for 24 h. At the beginning of the experiment bacterial culture growing for 24 h was used. From this bacterial culture, starting culture with OD 0.1 (104 cells per mL) was prepared; (**B**) The growth of cadmium(II) ions treated bacterial cells expressed as OD (absorbance measured at 605 nm) measured at the end of the treatment; in inset: the dependence obtained within the concentration range from 0 to 30 μg of Cd(II) per mL. Growth of the cultures was determined fully automatically for 24 h at 37 °C without shaking of each culture (n = 3).

At the beginning of the experiment bacterial culture growing for 24 h was used. From this bacterial culture, starting culture with OD 0.1 (10^4^ cells per mL) was prepared. A marked increase in growth of *S. aureus* without the presence of Cd(II) was detected and this is shown in Figure 4(A).

Due to the additions of different concentrations of cadmium(II) ions, start of the exponential growth phase and lag phase were shifted. The applied concentration of Cd(II) (50 μg·mL^−1^) was significantly inhibitory for the bacterial culture of *S. aureus* used in this study. In addition, we plotted the growth expressed as OD (absorbance measured at 605 nm) measured in the end of the treatment (24 h). The obtained dependence is shown in Figure 4(B). The resulting changes in the intensity of growth shown in Figure 4(B) are similar to those for growth inhibition of *S. aureus* bacterial strain RN4220 [76]. A mathematical model for the whole tested concentration range was used to describe dependence shown in Figure 4(B). The model had the following equation: y = 115.16e^−0.106x^, R^2^ = 0.932. In the concentration range from 0 to 30 μg of Cd(II) per mL, the dependence of the linear nature with the following equation y = −2.0537*x* + 70.016, R^2^ = 0.9641 was obtained and is shown in inset Figure 4(B). The obtained experimental data clearly shows that the bacterial culture used is very sensitive to the applied concentration of Cd(II) at levels greater than 30 μg·mL^−1^ and is well suited to assess environmental contamination by heavy metals.

**Figure 5.**
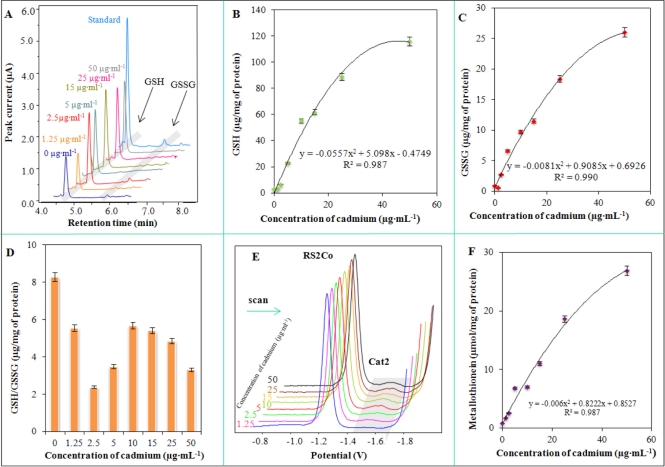
(**A**) HPLC-ED chromatograms of cell lysates of *S. aureus* treated with cadmium(II) ions measured at 900 mV. Changes in the content of (**B**) GSH and (**C**) GSSG, and (**D**) GSH/GSSG ratio in cadmium(II) ion-treated *S. aureus*; (**E**) DP voltammograms of MT isolated from treated *S. aureus*; (**F**) Dependence of MT and MT-like proteins levels on the applied cadmium(II) ions concentration. Other experimental conditions are given in Figure 4 and in the Experimental section.

Our aim was to characterize the bacterial culture used with regard to its potential application in biosensors. Therefore, we also studied a group of biologically important molecules. Molecules connected with thiol (–SH) metabolism were investigated first. Levels of GSH, GSSG and GSH/GSSG were monitored using well established high-performance liquid chromatography with electrochemical detection. Typical chromatograms of cell lysates of *S. aureus* treated with cadmium(II) ions are shown in Figure 5(A). GSH was detected at a retention time (RT): 4.8 min. and GSSG at RT = 5.9 min. Both signals were well separated and readily detectable. The amount of GSH increased with the increasing concentration of applied cadmium(II) ions linearly up to 20 μg·mL^−1^. After that the changes were more gradual and the course of the obtained dependence well fit with the polynomial model: y = −0.0557*x*^2^ + 5.098*x* − 0.4749, R^2^ = 0.987 (Figure 5(B)). Similarly, GSSG levels were changed, but GSSG level does not change in an organism without oxidative stress. It clearly follows from the results obtained that there are apparent dramatic increases in GSSG levels with the increasing applied concentration of cadmium(II) ions according to the following equation: y = −0.0081*x*^2^ + 0.9085*x* + 0.6926, R^2^ = 0.990 (24 h long cultivation, Figure 5(C)). Monitoring of oxidative stress in *S. aureus* is a very good indicator of environmental pollution with heavy metals. Moreover, GSH/GSSG ratio is also good indicator. From the point of view of the importance of both GSH and GSSG, the ratio above 9 shows good redox pool maintenance. Decreases of this ratio are a good indicator of oxidative stress in a cell and, therefore, indicates also the presence of some xenobiotics. It is not surprising that a ratio lower than 4 falls within the area of oxidative stress and lower than 2 means high oxidative stress with membrane damage and alteration of other biological functions (Figure 5(D)).

Metallothioneins (MT) as major proteins with heavy metals detoxification functions play an important role in protective mechanisms [65,78,79]. Typical Brdicka voltammograms of MT and MT-like proteins determined in *Staphylococcus aureus* are shown Figure 5(E). The catalytic signal called Cat2, which is used for quantification of proteins of interest [65,80–84], was detected at −1.55 V. The height of this peak was enhanced with the applied concentration of cadmium(II) ions according to the following equation: y = −0.006*x*^2^ + 0.8222*x* + 0.8527, R^2^ = 0.987.

**Figure 6.**
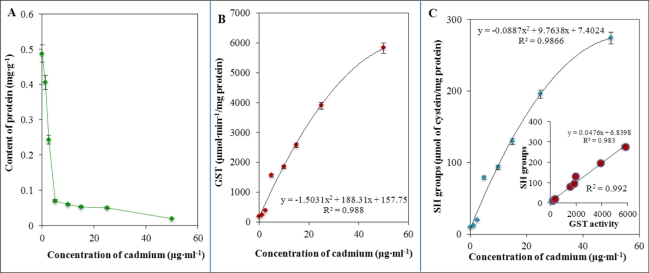
Spectrophotometric determination of (**A**) total proteins content by the Biuret method, (**B**) GST activity and (**C**) concentration of free –SH moieties; in inset: correlation between GST activity and free –SH moieties concentration. Other experimental conditions are given in Figure 4 and in the Experimental section.

Total proteins content directly corresponds to the activity of individual cells. In Figure 6(A) the dramatic decrease in total protein content at an applied concentration of cadmium(II) ions as low as 5 μg·mL^−1^ is clearly indicated. This decrease can be clearly associated with alteration of bacterial growth and, thus, with damage to some important biochemical pathways. Besides total proteins content, glutathione is a very important detoxifying molecule and must be activated by the enzyme called glutathione-S-transferase (GST). GST activity exhibited a behaviour similar to other thiol compounds, which means that the activity was enhanced with the increasing applied concentration of cadmium(II) ions according to the following equation: y = −1.5031*x*^2^ + 188.31*x* + 157.75, R^2^ = 0.988. The dependence is shown in Figure 6(B). Therefore, we also aimed to determine free –SH moieties. Their concentration was also enhanced with the increasing applied concentration of cadmium(II) according to the following equation: y = −0.0887*x*^2^ + 9.7638*x* + 7.4024, R^2^ = 0.987. The dependence is shown in Figure 6(C). Moreover, we attempted to correlate GST activity and concentration of free –SH moieties, which is shown in the inset in Figure 6(C). A strong correlation between these markers (R^2^ = 0.992) was found. Table 3 presents the correlation coefficients between levels of thiols. A strong link between the content of MT, GST, GSSG and –SH moieties was revealed. Correlation analysis revealed a strong positive correlation, because the correlation coefficients was always higher than R^2^ = 0.990. This can be clearly associated with simultaneous triggering of protective mechanisms due to the stress caused by cadmium(II) ions.

**Figure 7.**
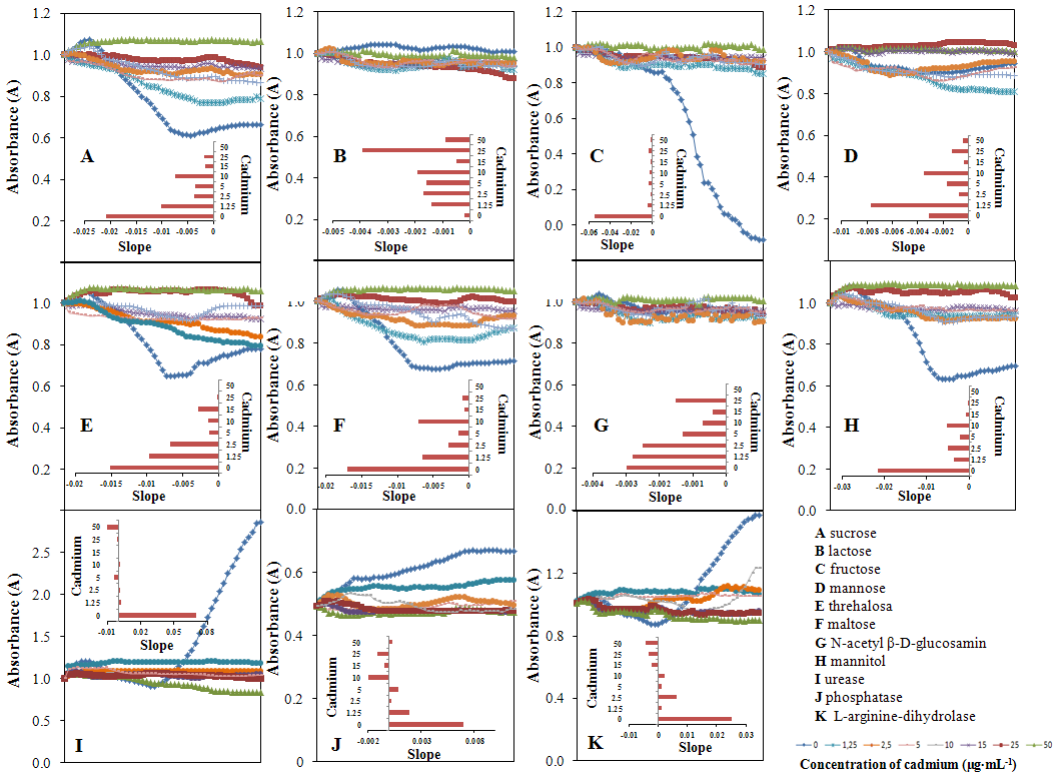
Spectrophotometric detection of *S. aureus* metabolism through determination of (**A**) sucrose; (**B**) lactose; (**C**) fructose; (**D**) mannose; (**E**) threalose; (**F**) maltose; (**G**) N-acetyl β-d-glucosamine; (**H**) mannitol; (**I**) urease; (**J**) phosphatise; and (**K**) l-arginine-dihydrolase; in inset: slopes of the liner mathematical model. Other experimental conditions are given in Figure 4 and in the Experimental section.

Monitoring of selected metabolic parameters was also an aim of this study. We examined sucrose, lactose, fructose, mannose, threalose, maltose, N-acetyl β-d-glucosamine, mannitol, urease, phosphatase and l-arginine-dihydrolase levels and the results are shown Figure 7(A–K), respectively.

Bacterial culture (100 μL, OD_605_ 0.1) was pipetted into the wells of plates (STAPHYtest, Lachema). Then, cadmium(II) ions were added in the following final concentrations (0, 1.25, 2.5, 5, 10, 15, 25 and 50 μg mL^−1^). Concentration of the metabolites of interest was determined at 15 min intervals for 24 h. Then, these dependencies were plotted and the slopes of the linear regressions are shown in the insets in Figure 7. The main group of these parameters represent changes in sugar metabolism (sucrose, lactose, fructose, mannose, threhalose, malthose, N-acetyl-d-glucosamine, mannitol) and three enzymes: urease, phosphatase and l-arginine-dihydrolase. Marked differences were observed in mannose, malthose and mannitol metabolism (Figure 7). Similarly, changes were observed in the activity of urease. Some changes in sucrose, fructose, trehalose, phosphatase and l-arginine dihydrolase were also measured, but these can be used for biosensing only in limited intervals. Lactose and N-acetyl-d-glucosamine showed no significant changes and these markers are certainly not suitable for use in sensors (Figure 7).

## 4. Conclusions

Metal ions that tend to accumulate in organisms is one of the greatest problems associated with heavy metals in the environment. Heavy metals can be thus accumulate through the food chain with the top being represented by predators. Food chain networks in sea are one of the most highly damaged environments. Coastal fish (such as the smooth toadfish) and seabirds (such as the Atlantic puffin) are often monitored for the presence of such contaminants. Biosensors help to monitor food safety and quality and to detect environmental pollution. In this study, the obtained experimental data provides basic information on the possible use of a range of biomolecules produced by a bacterial cell (*S. aureus* strain) as markers of metal pollution together with the fact that *S. aureus* can be employed as part of a biosensor. This paper is mainly aimed at the finding of the appropriate low molecular mass compounds, which are produced in higher or lower concentrations in bacteria as a response to stress caused by the presence of heavy metal ions. The results can be used for sensing of these toxic ions by combining spectrometry and/or electrochemistry and living bacteria. These types of sensors are of great interest because of the possibility of on-line monitoring they offer.

## Figures and Tables

**Table 1.** Calibration curves of cadmium(II) ions measured at various values of pH.

**pH**	**Equation**	**R^2^**	**n**	**R.S.D**	**LOD**	**LOQ**
pH 4	y = 34.478*x* − 11.073	0.994	5	4.6%	0.10 nM	0.40 nM
pH 5	y = 39.723*x* − 24.233	0.996	5	3.2%	0.01 nM	0.03 nM
pH 6	y = 32.108*x* + 17.833	0.998	5	5.1%	0.25 nM	1.00 nM

**Table 2.** The effect of cultivation medium additions into supporting electrolyte on cadmium(II) ions peak height.

**Sample**	**Linear model**	**R^2^**	**n**	**R.S.D (%)**	**Polynomial model**	**R^2^**
**Acetate buffer**	y = 39.049*x* − 20.494	0.9970	5	0.95	y = 0.0298*x*^2^ + 36.342*x* − 4.9257	0.9973
**Add 10% medium**	y = 32.935*x* + 43.672	0.9892	5	1.35	y = −0.0505*x*^2^ + 37.517*x* + 17.321	0.9903
**Add 50% medium**	y = 20.614*x* − 0.1286	0.9875	5	1.88	y = −0.0681*x*^2^ + 26.797*x* − 35.691	0.9929
**Add 80% medium**	y = 17.546*x* + 36.594	0.9882	5	2.49	y = −0.0791*x*^2^ + 24.724*x* − 4.6941	0.9981
**Add 100% medium**	y = 17.663*x* + 29.924	0.9915	5	3.10	y = −0.0702*x*^2^ + 24.04*x* − 6.7551	0.9993

R^2^ confidence coefficient, n number of independent measurements, R.S.D. relative standard deviation (%).

**Table 3.** Correlation coefficients between levels of MT, GST, GSSG and –SH moieties determined in *S. aureus* treated with cadmium(II) ions.

	**GST**	**−SH**	**GSSG**	**GSH**
MT	0.994	0.994	0.992	0.911
GST	x	0.992	0.993	0.907
−SH	x	x	0.997	0.940
GSSG	x	x	x	0.943

p = 0.05, n = 3.
